# Resuscitative endovascular balloon oclusion of the aorta (REBOA) in non-traumatic cardiac arrest in a pig-model: Influence on Return of spontaneous circulation (ROSC) with short-term survival, gas exchange and hemodynamics

**DOI:** 10.1371/journal.pone.0330346

**Published:** 2025-09-02

**Authors:** Randi Katrin Manegold, Philip Scheene, Andreas Wissmann, Fadi Al Rashid, Birte von der Beck, Nikolaus Pizanis, Clemens Kill, Joachim Risse

**Affiliations:** 1 Center of Emergency Medicine, University Hospital Essen, Essen, Germany; 2 Central Animal Laboratory, University Hospital Essen, Essen, Germany; 3 Clinic for Cardiology and Angiology, University Hospital Essen, Essen, Germany; 4 Clinic for Thoracic and Cardiovascular Surgery, University Hospital Essen, Essen, Germany; UT Health San Antonio: The University of Texas Health Science Center at San Antonio, UNITED STATES OF AMERICA

## Abstract

**Background:**

Survival of out-of-hospital cardiac arrest (OHCA) remains poor even when bystander cardiopulmonary resuscitation (CPR) with chest compression is initiated. Chest compressions provide only reduced cardiac output with limited perfusion of heart and brain and therfore may not avoid both death or poor neurological outcome in prolonged CPR.

We investigated the impact of resuscitative endovascular balloon occlusion of the aorta (REBOA) on hemodynamics, gas exchange and return of spontanous circulation (ROSC) with short-term survival during mechanical CPR (mCPR) with chest compression synchronized-ventilation (CCSV) in an atraumatic pig model.

**Methods:**

The study was performed on 20 pigs under general anaesthesia. REBOA catheter was placed in the thoracic aorta at the level of diaphragm beforecardiac arrest (CA) with ventricular fibrillation (VF) was induced. After 3 minutes of CA mCPR was started, CCSV was initiated at t = 5 min. Radomization to REBOA or control group, at t = 7 min inflation of REBOA ballon. CPR was continued until t = 18 min including defibrillation and intravenous epinephrine.

Primary endpoint was ROSC with short-term survival, secondary endpoints mean arterial pressures (MAP) and arterial bloodgas analyses.

**Results:**

ROSC was observed in n = 5 (REBOA) versus n = 1 (control) out of 10 animals, p = 0.141. All these animals remained stable for over an hour and thus also met the criteria for short-term survival. In the REBOA group, MAP was significantly increased following blockage of the ballon. Arterial blood gas analyses (ABG) showed a trend to higher PaO_2_ (REBOA 375 ± 147 mmHg vs control 277 ± 129 p = 0,220), higher pH-value (REBOA 7,37 ± 0,06 vs control 7,24 ± 0,12 p = 0,052) and less increased PaCO_2_ (REBOA 38 ± 7 mmHg vs control 59 ± 21 mmHg p = 0,056) at t = 14 min.

**Conclusion:**

In our animal resuscitation model of non-traumatic CA, REBOA showed a significant increase in MAP and a favourable influence on gasexchange, associated with a trend towards higher ROSC rates and short-term survival. It remains to be seen whether these effects can be replicated in larger experimental and clinical studies.

## Introduction

Every year in Europe, around 50–100 people per 100 000 inhabitants suffer OHCA. Of these patients, only 8% achieve a stable return to spontaneous circulation and are discharged from hospital alive [1]. Even fewer achieve a complete or almost complete neurological recovery and satisfactory long-term survival [2,3]. Despite all medical progress, these results have remained poor for decades. Similar figures are available from the USA and Asia [4–6]. Organ damage caused by ischemia and reperfusion – especially of the brain – plays a major role. Chest compression as the only commonly available initial intervention is unlikely to achieve the cardiac output required for adequate tissue oxygenation. This is why the circulatory situation under manual chest compression is often referred to as “low-flow”. The literature varies as to what cardiac output is achieved (7,8% −46%) and how it is best measured. Coronary blood flow is often even more severely reduced [7–10]. There is no doubt that long low-flow times, be it 10 or 50% of a normal cardiac output, are associated with poor outcome. It leads to post-resuscitation syndrome with all the negative consequences such as reactive cerebral hyperaemia and vasoplegia [11,12]. When mechanical cardiopulmonary resuscitation (mCPR) in professional CPR is used, the cardiac output may be slightly higher, but an improvement in outcome has not yet been demonstrated [13]. Resuscitative endovascular balloon occlusion of the aorta (REBOA) could be an approach to improve blood flow and thus the oxygen supply to the critical organs heart and brain during cardiopulmonary resuscitation. But it is not just about perfusion. Experience shows that even prehospital resuscitated patients who show adequate blood pressures and sufficient end-tidal carbondioxid (ETCO_2)_ during CPR often have severe gas exchange disorders in the first ABG. Here too, with a focus on ventilation and gas exchange, further research is urgently needed.

REBOA was first used to control critical bleeding in the lower half of the body in 2011 by British vascular surgeons for severe trauma in context of war injuries [14]. Since then, it has been considered the last resort in severe hemorrhagic shock, e.g., after severe penetrating trauma [15,16] or obstetric complications [17,18]. Most recently, a randomized controlled trial (RCT) for trauma patients in England showed no survival advantage and possibly even a disadvantage for the REBOA group [19], but even these results are being discussed quite controversially [20]. In the non-trauma setting experience in humans is very limited, data from animal experiments are varying and show positive effects on various haemodynamic parameters and not always on the ROSC rate [21–23]. As the pathophysiology of non-traumatic circulatory arrest is fundamentally different from that of haemorrhagic shock, more differentiated investigation is necessary. In 2022, a research group in China was able to achieve a very high ROSC rate in a pig experiment with the combination of REBOA and chest compression synchronized ventilation (CCSV) [24].

The aim of this study was to shed more light on the effects of REBOA on hemodynamics and gas exchange, in addition to influencing the ROSC rate and short-term survival, in order to gain a deeper understanding of the physiology under REBOA-therapy. In this experiment, we used the CCSV ventilation mode, which has been shown to achieve higher MAPs by increasing the intrathoracic pressure during compression [25], so that a synergistic effect with REBOA is conceivable. We also wanted to focus on the aspect of gas exchange and investigate the hypothesis that the upper half of the body is not only perfused with a higher MAD, as a few studies have already shown, but also with better oxygenated and decarboxylated blood.

## Materials and methods

This study was approved by the local authority for animal protection (LANUV, 81–02.04.2021.A299), planned and carried out in accordance with the ARRIVE criteria and preregistrated at animaltestinfo.de (NTP-ID: 00045715-2-5). The study was performed on twenty healthy female domestic pigs (Sus scrofa domestica), aged 3–5 months, weighing 37,6 ± 2,5 kg. Animals were obtained by a local farmer, which is consistent with §19 of the German animal welfare ordinance. All animals received humane care in compliance with the Institutional Animal Care and Use Committee guidelines.

The animals are kept in special, air-conditioned animal husbandry rooms for pigs in accordance with EU Directive 2010/63. The pigs are kept in groups of at least two animals. The floor has a non-slip epoxy resin coating and is sprinkled with straw. As enrichment, the animals may receive a hay basket, a permanently mounted wall brush and balls or other chewing equipment in regular rotation per box. The animals are kept at room temperature of appr. 18° C and receive restricted standard food and water ad libitum. After a familiarization period of at least 10 days, the animals are used for the experiments. The animals are cared for by trained animal keepers before the experiment is initiated and acclimatized to a member of the research group during the acclimatization period. All members of the research group attended and successfully completed the required courses in laboratory animal science. Specialised veterinarians in laboratory animal science were avaliable in the laboratory at all times in case of complications.

### Preparation of the test animals

Prior to the experiment, the animals were given 12 hours off food with free access to water. On the day of the experiment, the animals were premedicated one hour before the start of the experiment with ketamine („Ketamin O.K.® 50mg/5ml solution for injection“Rotexmedica GmbH, Germany) 30 mg/kg body weight (BW), 0.02-0.05 mg/kgBW atropine („Atropinsulfat® B. Braun 0,5mg/ml solution for injection “B. Braun AG, Germany) and 2 mg/kgBW azaperone („Stresnil ad us. Vet. ® 40mg/ml solution for injection “Elanco Deutschland GmbH, Germany), and after reaching an adequate sedation depth an intravenous cannula („Vasofix ® Safety Kanüle, 20G“B. Braun AG, Germany)was inserted into an ear vein. Vital signs monitoring starting with peripheral oxygen saturation and cagnography was set up („Corpuls3 ®“corpuls - GS Elektromedizinische Geräte G. Stemple GmbH, Germany). After administration of 2-3 mg/kgBW propofol („Propofol-®Lipuro 20mg/ml solution for injection “ B. Braun AG, Germany) and fentanyl 2-3 µg/kgBW („Fentanyl-hameln® 500µg/10ml solution for injection Hameln Pharma GmbH, Germany) endotracheal intubation was performed (ID 6.0mm). To maintain anaesthesia, propofol was infused at approx. 7mg/kg*h, midazolam (“Midazolam- ratiopharm® 5mg/5ml solution for injection”, ratiopharm GmbH, Germany) up to 0.75 mg/kgKG and fentanyl at 15 µg/kg/h as required. The animals were fully relaxed with 1 mg/kg/KG rocuronium („Rocuronium® B. Braun, 50 mg/5 ml solution for injection “B. Braun AG, Germany) under total intravenous anesthesia and volume-controlled ventilation with Vt = 10 ml/kg BW, PEEP 5 mbar and adjustment of the respiratory rate according to Et CO_2_ („Dräger Fabius Trio®“Dräger AG, Germany).

Monitoring included ECG, pulse oximetry, capnography and body temperature (via a rectal temperature probe) during instrumentation. An arterial cannula („Arrow® Radial Access Sheath Kit“ Teleflex Medical GmbH, Germany) was inserted into the common carotid artery and a 3-lumen central venous catheter („ARROWg + ard Blue®“ Teleflex Medical GmbH, Germany) into the internal jugular vein using the Seldinger technique and sonographic control. Hemodynamic pressures were recorded continuously („Combitrans Haemofix®“ B. Braun AG, Germany).

A catheter sheath („Arrow® Percutaneous Sheath Introducer – 6 French“ Teleflex Medical GmbH, Germany) was placed via the right jugular vein for insertion of a transvenous pacemaker („Arrow® Temporary Pacing Kit – Balloon Flotation Catheter – 5 French“ Teleflex Medical GmbH, Gemany). A catheter sheath was also inserted into a femoral artery („Arrow® Percutaneous Sheath Introducer – 7.5 French “ Teleflex Medical GmbH, Germany), into which the REBOA system („ER- REBOA®“ Prytime Medical Devices Inc, USA) was then inserted and advanced 2 cm above the level of the diaphragm corresponding to zone 1 (defined as from origin left subclavian artery to celiac trunk). The position was determined by external landmarks and measurement with a tape measure. Before starting the experiment, the balloon was blocked for 30 s and the correct functioning was verified by an increase in MAP of > 20 mmHg.

Defibrillator patches („Corpuls® corpatch easy“ corpuls – GS Elektromedizinische Geräte G. Stemple GmbH, Germany) were placed in a lateral-lateral position, connected to the defibrillator (Corpuls C3, corpuls – GS Elektromedizinische Geräte G. Stemple GmbH, Germany) unit and an additional ECG lead was established via the patches.

The animals were fixed in a special holder in supine position and the plunger of the mechanical resuscitation device„corpuls cpr®“ corpuls – GS Elektromedizinische Geräte G. Stemple GmbH, Germany) was adjusted.

Following a 15–20 minute period to achieve steady state with roomair and a target for PaCO_2_ = 35–40 mmHg/5,3 kPA 1 mg/kgBW rocuronium was given and experimental procedures were started.

### Experimental procedures

**Fig 1:**
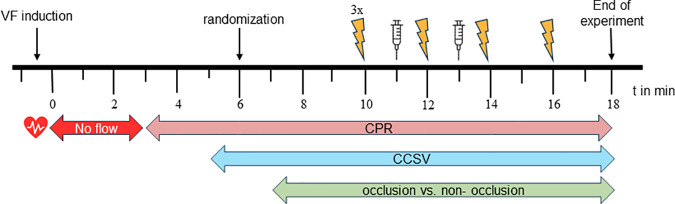
Diagram of the experimental procedures. Measures related to the time (min). VF = ventricular fibrillation, CPR = cardiopulmonary resuscitation, flash symbol = defibrillation with 200J, syringe symbol: injection of 1 mg epinephrine.

Ventricular fibrillation (VF) was induced with a right ventricular paced electrode and AC 7.5 to 15V. VF remained untreated for 3 minutes without any ventilation or chest compression. At t = 3 min mCPR was started without ventilaion (compression only CPR). At t = 5 min mechanical ventilation in CCSV mode was applied with an emergency and transport ventilator (MEDUMAT Standard2, Fa. Weinmann GmbH + Co.KG, Germany) with a setting of pmax = 60mbar, Tinsp 205ms, Respiratory rate = 100/min. Randomization out using a sealed envelope followed and at t = 7 min and the balloon in the REBOA group was blocked with 10 ml NaCl while it remained empty in the control group. At t = 10 min, the first rhythm analysis was performed with 3 consecutive biphasic defibrillations of 200 J each. From then on, a rhythm analysis was performed every 2 minutes followed by defibrillation if a shockable rhythm was detected (t = 12 min, t = 14 min, t = 16 min). At t = 11 min and t = 13 min, 1 mg epinephrine was administered via the central venous line. Arterial and venous blood gas analyses were performed at time points t = −2 min, t = 4 min, t = 7 min, t = 10 min und t = 14 min. Vital signs were recorded continuously. In case of ROSC, the animal was further treated according to the follow-up protocol and euthanized after 60 min under anesthesia using potassium cholride. ROSC was defined by the derivation of a stable arterial pressure curve with a systolic blood pressure > 50 mmHg for at least 30 s via the arterial line in the carotid artery. In the absence of ROSC, the experiment was terminated at time t = 18 min after the rhythm analysis that followed the 4th shock. Fig 1 summarizes the chronological sequence of interventions during the experiment.

The animals were anesthetized at the beginning of the experiment and euthanized under anesthesia at the end of the experiment. The procedure took about 3–5 hours from induction of premedication to euthanasia, depending on how much time the instrumentation took and wether a follow-up was carried out after ROSC. The animal was under general anesthesia at all times. It is therefore a final experiment in which the animals did not wake up again and no suffering or pain was expected. Humane endpoints were not required.

### Data analysis and statistics

A power analysis was conducted using GPower (Version 3.1.9.6, Heinrich Heine University Düsseldorf, Germany) based on a two-tailed* t*-test with an alpha of 0.05 and a beta of 0.20, as commonly applied in large animal studies (Dogan et al., 2020; Kill et al., 2015; Kill et al., 2014). Data from Dogan et al. (2019), which assessed correct REBOA positioning, indicated a biologically relevant difference of ~150% based on hemodynamic differences in blood pressure between groups. With a standard deviation of 1.3, this yielded an effect size of 1.2. Assuming equal group allocation (ratio 1:1), the required sample size was calculated to be* n = 20.“

All data were processed using Excel software (Version 2017, Microsoft Corporation, Redmond, WA, USA) and IBM SPSS Statistics (Version 27.0, IBM Corporation, Armonk, NY, USA).

Primary endpoint was ROSC with follow up to short-term survival, secondary endpoints mean arterial pressures (MAP) and arterial bloodgas analyses.

Due to the small group size, a Fisher exact test was used to calculate the significance of the primary endpoint.

The mean arterial blood pressure (MAP) was obtained by integrating the area-under-cruve in 10-seconds interval of the according timepoints. Data were tested for normal distribution using the Shapiro-Wilk test and visualized with quantile-quantile (Q-Q) plots. Homogeneity of variances was assessed using Levene’s test. Depending on the results, group comparisons were conducted using either an independent two-sample t-test (for normally distributed, homoscedastic data) or Welch’s t-test (for normally distributed, heteroscedastic data). In addition, a one-way analysis of variance (ANOVA) with repeated measures was performed.

Similar to the blood pressure parameters, the measurement results of the blood gas analysis were also analyzed for normality using the quantile-quantile plot and Kolmogorov-Smirnov residual.

## Results

A total of 20 pigs were included according to the protocol described above. There were no differences between the control group and the REBOA group regarding to basic characteristics and baseline parameters of haemodynamic and gasexchange (Table 1).

**Table 1:** Baseline parameters of the two groups before the start of the test.

*Parameter*	*Group*		*P*
	*REBOA*	*Control*	
n	10	10	
weight (kg)	37,6 ± 2,5	36,2 ± 2,24	0,189
female sex	100%	100%	
temperature (°C)	36,6 ± 0,44	36,4 ± 0,42	0,222
heart rate (bpm)	101 ± 25,7	88 ± 10,12	0,353
systolic bloodpressure (mmHg)	103 ± 10,21	104 ± 7,96	0,683
diastolic bloodpressure (mmHg)	66 ± 11,28	69,3 ± 8,37	0,466
pH	7,458 ± 0,04	7,476 ± 0,05	0,378
pCO_2_ (mmHg)	41,91 ± 4,79	40,96 ± 4,78	0,663
pO_2_ (mmHg)	110,8 ± 20,1	117,2 ± 21,6	0,505
lactate (mmol/l)	2,97 ± 1,62	2,69 ± 1,52	0,691

Parameters given in mean ±SD, p = statistical significance by two-sample t-test. No differences between the two groups.

### ROSC rates with short-term survival in the REBOA and control groups

With regard to the primary endpoint ROSC rate, the experiments showed the following result: In the REBOA group 5 out of 10 pigs achieved ROSC, the control group 1 out of 10 pigs (Table 2). Thus, the ROSC rate in the REBOA group appears to be higher, the difference did not reach statisticall significance according to the Fisher Exact Test (p = 0.141).

**Table 2:** Rate of Return of Spontanous Circulation (ROSC) in Resuscitative endovascular balloon occlusion of the aorta (REBOA) and control group.

	*Group*	
	*REBOA*	*control*
*ROSC*	*5 (50%)*	*1 (10%)*
*no ROSC*	*5 (50%)*	*9 (90%)*
*Fisher exact test*	*p = 0,141*	

p = statistical significance in Fisher exact test.

All of the successfully resuscitated animals achieved ROSC within the first shock delivery series after t = 10 min and before administration of epinephrine. The one animal in the control group was successfully defibrillated with the first shock of the series of three. Of the five animals in the REBOA group, three were converted with the first shock and one each with the second and third shock.The pigs that achieved ROSC remained stable during the one-hour follow-up period and during gradual unblocking of the balloon under moderate volume and catechoalmin therapy. This allows us to determine the same result for short-term survival as for the primary endpoint ROSC rate: one in ten pigs survived one hour in the control group and five in ten in the REBOA group (Table 3).

**Table 3:** Rate of short-term survival in Resuscitative endovascular balloon occlusion of the aorta (REBOA) and control group.

	*Group*	
	*REBOA*	*control*
*short-term-survival*	*5 (50%)*	*1 (10%*
*no short-term survival*	*5 (50%)*	*9 (90%)*
*Fisher exact test*	*p = 0,141*	

p = statistical significance in Fisher exact test.

### Influence of REBOA on the hemodynamics

**Fig 2:**
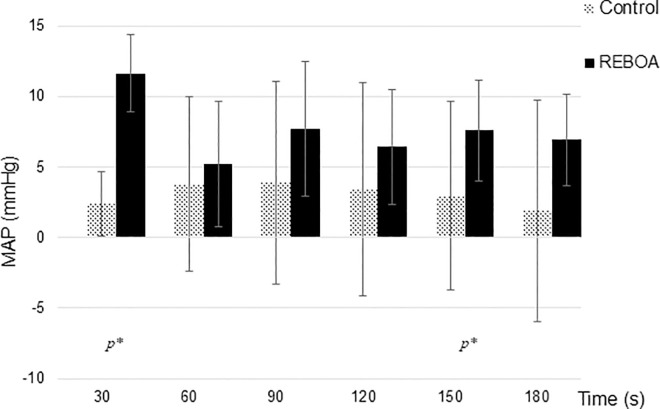
Mean increase in Mean arterial pressure (MAP) after block of the Resuscitative endovascular balloon oclusion of the aorta(REBOA)- catheter. Pressures compared to baseline pressure during conventional cardiopulmonary resuscitation (CPR), given in mean ± SD (in mmHg), p* = statistical significance in two-sample t-test.

The arterial pressure was recorded continuously. All measured values refer to the mean arterial blood pressure (MAP), i.e., the integral between systolic and diastolic blood pressure at a fixed time interval of 10 seconds. For example, if a blood pressure is given at t = 30 s, this refers to the average MAP recorded 10 seconds after the time t = 30 s. The baseline MAP two minutes before the start of the test did not differ between the groups and was on average 82.5 mmHg (SD ± 8.6 mmHg) in the control group and 79.2 mmHg (SD ± 11.45 mmHg) in the REBOA group (p = 0.478 in the 2-sample t-test). After induction of VF we saw a drop in the mean MAP to almost zero in both groups. The MAP immediately after the start of chest compressions was 36.4 mmHg (± 15.2 mmHg) in the control group and 33.5 mmHg (SD ± 5.6 mmHg) in the REBOA group (p = 0.581 in the 2-sample t-test). 30 s after the start of CCSV ventilation, the pressures continued to show no significant differences between the groups. These increases in blood pressure demonstrate the quality of the resuscitation measures. Fig 2 shows the increase in MAP after blocking the balloon. This is higher in the REBOA group than in the control group with statistical significance after 30 s and 150 s.

In summary, it can be stated that there is a clear effect on the MAP after the balloon is inflated. This effect is independent of administration of epinephrine.

### Influence of REBOA on gas exchange

**Fig 3:**
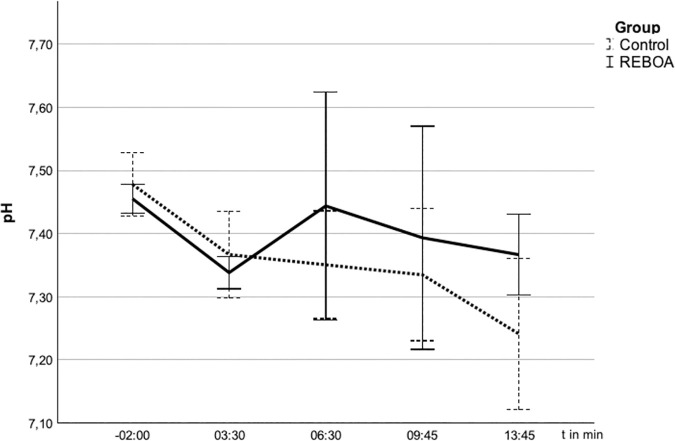
pH in the arterial blood gas analyses (ABG) after blocking of the balloon at t = 0. 3 The pH over time (s) given in mean ± SD. The pH falls more sharply in the control group and remains within the normal range in the REBOA group.

**Fig 4:**
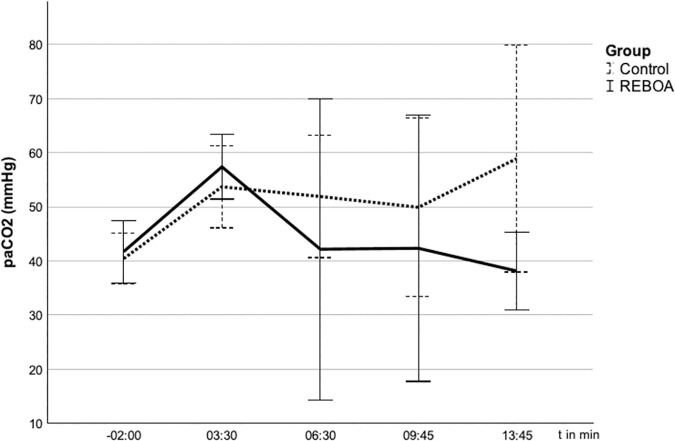
Arterial carbon dioxide partial pressure (pCO_2_) in the arterial blood gas analyses (ABG) after blocking of the balloon at t = 0. 4 The pCO_2_ (mmHg) over time (s) given in mean ± SD. The pCO_2_ rises in the control group and falls in the REBOA group.

**Fig 5:**
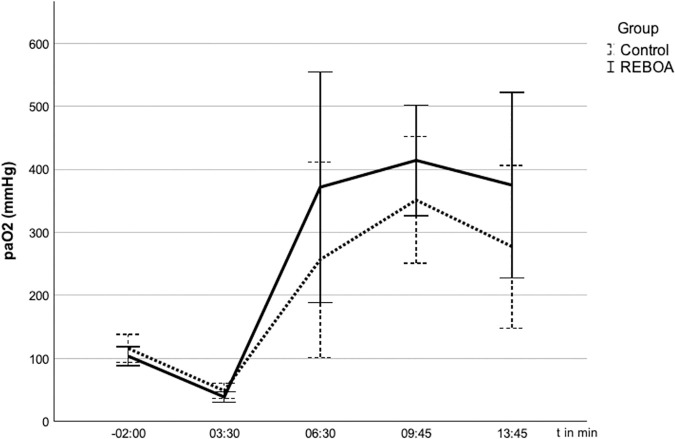
Arterial oxygen partial pressure (pO_2_) in the arterial blood gas analyses (ABG) after blocking of the balloon at t = 0. 5 The pO_2_ (mmHg) over time (s) given in mean ± SD. The pO_2_ tends to be higher in the REBOA group.

Before the start of the experiment (t = −2 min), the pigs in both groups showed normal values of the gas exchange under ventilation in BIPAP mode with FiO_2_ 0.21 in blood samples obtained from the common carotid artery. After the start of the experiment, all animals developed hypoxemia at t = 3:30 min (REBOA PaO_2_ 46 + /- 17 mmHg, Control 47 + /- 12 mmHg) and hypercapnia (REBOA PaCO_2_ 54 + /-7 mmHg, Control 55 + /-9 mmHg) without significant differences between the groups. After blocking the balloon, pH, pCO_2_ and pO_2_ developed in the two groups as shown in Figs 3–5.

The oxygen partial pressure at the last measurement at t = 13.45 min in the REBOA group with 375 + /- 147 mmHg tends to be above that of the control group with 277 + /-129 mmHg. However, this difference did not reach statistical significance (p = 0,220).

The carbon dioxide partial pressure at the last measurement at t = 13.45 min in the REBOA group with 38,12 + /-7,21 mmHg tends to be below that of the control group with 58,90 + /-20,95 mmHg (p = 0056).

In the REBOA group, the pH remains in the noramal range with 7,37 + /- 0,06 while in the control group with 7,24 + /- 0,12 shows a relevant acidosis.These differences also did not reach statistical significance (p = 0,052).

### *Influence of REBOA on lactate and bicarbonate (HCO*_*3*_^*-*^)

**Fig 6:**
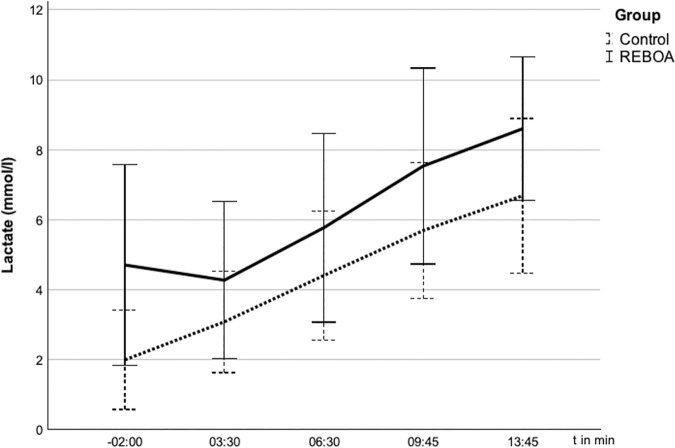
Lactate in the arterial blood gas analyses (ABG) after blocking of the balloon at t = 0. 6 Lactate (mmol/l) over time (s) given in mean ± SD. The lactate levels rise parallel in both groups from t = 03:30 min.

**Fig 7:**
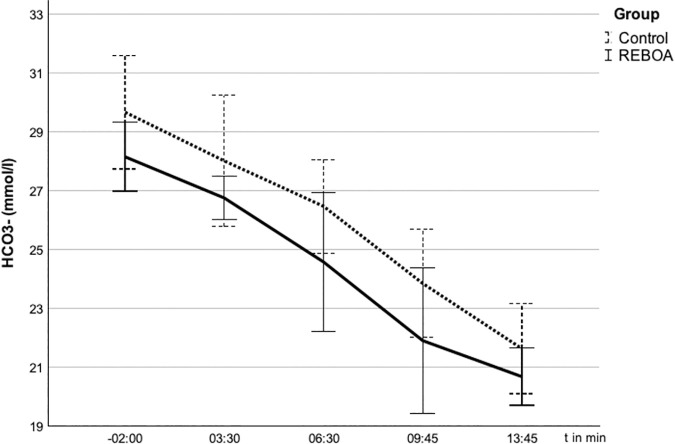
Bicarbonate (HCO_3_^-^) in the arterial blood gas analyses (ABG) after blocking of the balloon at t = 0. 7 HCO_3_^-^(mmol/l) over time (s) given in mean ± SD. The concentration of HCO_3_^-^ rises parallel in both groups.

The metabolic parameters of the BAG behaved as shown in Figs 6 and 7.

The average arterial lactate concentration at t = −2:00 was 1.7 mmol/l in the control group and 2.8 mmol/l in the REBOA group (p = 0.207) and increased during the experiment in both groups. The reference range for pigs is slightly higher than for humans and is given as up to 0.5 to 5.5 mmol/l, so that both groups were within the normal range at the start of the test. In the last measurement at t = 13:45, the lactate level reached a concentration of 6.7 mmol/l in the control group and 7.6 mmol/l in the REBOA group (p = 0.522). There were no statistically significant differences between the groups.

The average arterial HCO_3_^-^ concentration at t = −2:00 was 29.9 mmol/l in the control group and 29.2 mmol/l in the REBOA group (p = 0.408). At the beginning of the experiment, the bicarbonate levels in both groups were slightly above the normal range (reference value 22–26 mmol/l) without the administration of buffer substances. In the course of the experiment, they fell continuously in both groups down to 21.6 mmol/l in the control group and 20.7 mmol/l in the REBOA group at t = 13:45 (p = 0.282). There were no statistically significant differences in the respective groups.

## Discussion

In this experimental animal study we investigated the influence of REBOA on ROSC rate with short-term survival and hemodynamics during the early period of CPR. Due to the small number of animals the higher incidence of ROSC was not statistically significant. However, the significantly higher increase in MAP in the REBOA group and the better gas exchange support the assumption of a positive effect on the ROSC rate.

Admittedly, there are finer circulatory parameters such as coronary perfusion pressure (CPP) and cerebral perfusion pressure than global MAP. Nevertheless, MAP also has its place in resuscitation research and studies have been and continue to be carried out [26] to define blood pressure targets for during CPR and for post-resuscitation care. It has been shown that a higher MAP during CPR is associated with a higher ROSC rate [27]. As MAP is the parameter that can be measured most easily in everyday clinical practice, it is of interest for transferability to bedside. The second positive effect that could be shown are the PaCO_2_ values, that were in normal range or less increased compared to the control group, and also the pH value remained in the normal range for longer during cardiopulmonary resuscitation. Interestingly, the influence on gas exchange is greater than that on lactate and bicarbonate. Patients under resuscitation develop a mixed respiratory-metabolic acidosis usually very quickly. This leads to electrolyte shifts, reduces the effectiveness of epinephrine and jeopardizes the success of defibrillation [28]. Blood pH is an independent predictor for neurologic outcome after resuscitation [29]. Both, higher MAP and stable acid-base balance have a favorable influence on resuscitation success.

The main limitation of this study is the small group size. So even though there appears to be a clear tendency towards a higher ROSC rate in the REBOA group, this did not reach statistical significance. In the meantime, several study have been published that showed a significantly higher ROSC rate in pigs under REBOA in a similar experimental settings which supports the assumption of a positive effect. Most recently, Dogan et al found that both REBOA and IABP significantly increased the ROSC rate in the porcine experiment compared to mechanical chest compression alone [30]. Another limitation is the relatively short observation period. Since the pigs that achieved ROSC in our experiment already did so in the first defibrillation cycle, they were no longer available for the observation of MAP and gas exchange under REBOA, which further reduced the group size. In addition, the observation time was short overall. Since acidosis and very low blood pressure become problematic with longer duration of resuscitation, the REBOA effect may then become more pronounced.

Beyond, sophisticated resuscitation research is not based on ROSC and survival as a target value, but considers the neurological outcome as the gold standard. In a terminal trial like ours, it is not possible to make a statement on this.

Even though the pig model is an well established and necessary instrument in resuscitation research [31], the transferability of the results to humans must be critically examined. The highly standardized setting in the laboratory is only comparable with the reality of the emergency of sudden cardiac arrest to a limited extent. The short time intervals of this experiment can only be achieved in prehospital circulatory arrest in exceptional cases. The arterial sheath here was placed before the initiation of ventricular fibrillation. However, it has already been shown that REBOA in the prehospital setting is feasible [32].

We believe that the animal data which have been collected so far and to which we are making a very small contribution, justifies its use in humans in the context of controlled studies by specialized teams. The REBOARREST study is currently investigating the effect of REBOA in non-traumatic circulatory arrest in humans [33]. The results will advance the discussion. As with similar highly invasive procedures, e.g., ECLS, the main challenge is to precisely define the patient population that will benefit. The conflict here is that REBOA is in competition with ECLS, especially at these specialized centers. REBOA could potentially be a way of improving resuscitation outcomes precisely where ECLS is not available. Maybe the best conditions for the first human applications would be where femoral access already exists or can be easily established (IHCA in the cardiac catheterization lab, in intensive care units, in the OR). REBOA may not be the final solution, but the approach of redistributing cardiac output in favor of the upper half of the body is worth pursuing, as ROSC rate and gas exchange are significantly improved. Ideally, an even simpler method will be developed to use this mechanism. One could also imagine combining REBOA and ECLS and initially blocking aortally in case of delayed venous cannulation or other difficulties.

Our experiment and many REBOA studies focus on the very early phase and rapid ROSC. What urgently needs to be investigated further is how long it is possible to block without consequential damage and whether there are any positive effects at all later in the resuscitation process.

## Conclusion

Occlusion of the aorta during shockable circulatory arrest favorably influences the parameters MAP and paCO_2_, which could result in a higher ROSC rate, even though this experiment did not reach significance. It remains to be seen whether these effects can be replicated in larger experimental and clinical studies. The patient population and the settings in which REBOA could potentially be beneficial need further characterization.

## References

[1] Gräsner J-T, Wnent J, Herlitz J, Perkins GD, Lefering R, Tjelmeland I, et al. Survival after out-of-hospital cardiac arrest in Europe - Results of the EuReCa TWO study. Resuscitation. 2020;148:218–26. doi: 10.1016/j.resuscitation.2019.12.042 32027980

[2] Dillenbeck E, Svensson L, Rawshani A, Hollenberg J, Ringh M, Claesson A, et al. Neurologic recovery at discharge and long-term survival after cardiac arrest. JAMA Netw Open. 2024;7(10):e2439196. doi: 10.1001/jamanetworkopen.2024.39196 39392629 PMC11581594

[3] Amacher SA, Bohren C, Blatter R, Becker C, Beck K, Mueller J, et al. Long-term survival after out-of-hospital cardiac arrest. JAMA Cardiol. 2022;7(6):633. doi: 10.1001/jamacardio.2022.0795 35507352 PMC9069345

[4] Virani SS, Alonso A, Aparicio HJ, Benjamin EJ, Bittencourt MS, Callaway CW, et al. Heart disease and stroke statistics-2021 update: a report from the American Heart Association. Circulation. 2021;143(8):e254–743. doi: 10.1161/CIR.0000000000000950 33501848 PMC13036842

[5] Chin YH, Yaow CYL, Teoh SE, Foo MZQ, Luo N, Graves N, et al. Long-term outcomes after out-of-hospital cardiac arrest: a systematic review and meta-analysis. Resuscitation. 2022;171:15–29. doi: 10.1016/j.resuscitation.2021.12.026 34971720

[6] Yan S, Gan Y, Jiang N, Wang R, Chen Y, Luo Z, et al. The global survival rate among adult out-of-hospital cardiac arrest patients who received cardiopulmonary resuscitation: a systematic review and meta-analysis. Crit Care. 2020;24(1). doi: 10.1186/s13054-020-2773-2 PMC7036236 32087741

[7] Engoren M, Severyn F, Fenn-Buderer N, DeFrank M. Cardiac output, coronary blood flow, and blood gases during open-chest standard and compression-active-decompression cardiopulmonary resuscitation. Resuscitation. 2002;55(3):309–16. doi: 10.1016/s0300-9572(02)00214-9 12458068

[8] Kimmel E, Beyar R, Dinnar U, Sideman S, Kishon Y. Augmentation of cardiac output and carotid blood flow by chest and abdomen phased compression cardiopulmonary resuscitation. Cardiovasc Res. 1986;20(8):574–80. doi: 10.1093/cvr/20.8.574 3791346

[9] Marshall RA, Morton JS, Luchkanych AMS, El Karsh Y, El Karsh Z, Morse C, et al. Left ventricle chest compression improves ETCO2, blood pressure, and cerebral blood velocity in a swine model of cardiac arrest and cardiopulmonary resuscitation. Resusc Plus. 2022;12:100326. doi: 10.1016/j.resplu.2022.100326 36407570 PMC9672447

[10] Fodden DI, Crosby AC, Channer KS. Doppler measurement of cardiac output during cardiopulmonary resuscitation. J Accid Emerg Med. 1996;13(6):379–82. doi: 10.1136/emj.13.6.379 8947791 PMC1342801

[11] Nolan JP, Neumar RW, Adrie C, Aibiki M, Berg RA, Böttiger BW, et al. Post-cardiac arrest syndrome: epidemiology, pathophysiology, treatment, and prognostication. A Scientific Statement from the International Liaison Committee on Resuscitation; the American Heart Association Emergency Cardiovascular Care Committee; the Council on Cardiovascular Surgery and Anesthesia; the Council on Cardiopulmonary, Perioperative, and Critical Care; the Council on Clinical Cardiology; the Council on Stroke. Resuscitation. 2008;79(3):350–79. doi: 10.1016/j.resuscitation.2008.09.017 18963350

[12] Lemiale V, Huet O, Vigué B, Mathonnet A, Spaulding C, Mira J-P, et al. Changes in cerebral blood flow and oxygen extraction during post-resuscitation syndrome. Resuscitation. 2008;76(1):17–24. doi: 10.1016/j.resuscitation.2007.06.028 17714849

[13] Sheraton M, Columbus J, Surani S, Chopra R, Kashyap R. Effectiveness of mechanical chest compression devices over manual cardiopulmonary resuscitation: a systematic review with meta-analysis and trial sequential analysis. West J Emerg Med. 2021;22(4):810–9. doi: 10.5811/westjem.2021.3.50932 35353993 PMC8328162

[14] Stannard A, Eliason JL, Rasmussen TE. Resuscitative endovascular balloon occlusion of the aorta (REBOA) as an adjunct for hemorrhagic shock. J Trauma. 2011;71(6):1869–72. doi: 10.1097/TA.0b013e31823fe90c 22182896

[15] Brenner M, Bulger EM, Perina DG, Henry S, Kang CS, Rotondo MF, et al. Joint statement from the American College of Surgeons Committee on Trauma (ACS COT) and the American College of Emergency Physicians (ACEP) regarding the clinical use of Resuscitative Endovascular Balloon Occlusion of the Aorta (REBOA). Trauma Surg Acute Care Open. 2018;3(1):e000154. doi: 10.1136/tsaco-2017-000154 29766135 PMC5887776

[16] Bukur M, Gorman E, DiMaggio C, Frangos S, Morrison JJ, Scalea TM, et al. Temporal changes in REBOA utilization practices are associated with increased survival: an analysis of the AORTA registry. Shock. 2021;55(1):24–32. doi: 10.1097/SHK.0000000000001586 32842023

[17] Stensaeth KH, Sovik E, Haig INY, Skomedal E, Jorgensen A. Fluoroscopy-free Resuscitative Endovascular Balloon Occlusion of the Aorta (REBOA) for controlling life threatening postpartum hemorrhage. PLoS One. 2017;12(3):e0174520. doi: 10.1371/journal.pone.0174520 28355242 PMC5371310

[18] Manzano-Nunez R, Escobar-Vidarte MF, Naranjo MP, Rodriguez F, Ferrada P, Casallas JD, et al. Expanding the field of acute care surgery: a systematic review of the use of resuscitative endovascular balloon occlusion of the aorta (REBOA) in cases of morbidly adherent placenta. Eur J Trauma Emerg Surg. 2018;44(4):519–26. doi: 10.1007/s00068-017-0840-4 28929283

[19] Jansen JO, Hudson J, Cochran C, MacLennan G, Lendrum R, Sadek S, et al. Emergency department resuscitative endovascular balloon occlusion of the aorta in trauma patients with exsanguinating hemorrhage: the UK-REBOA randomized clinical trial. JAMA. 2023;330(19):1862–71. doi: 10.1001/jama.2023.20850 37824132 PMC10570916

[20] Brede JR, Rehn M. The end of balloons? Our take on the UK-REBOA trial. Scand J Trauma Resusc Emerg Med. 2023;31(1):69. doi: 10.1186/s13049-023-01142-5 37908007 PMC10619299

[21] Tiba MH, McCracken BM, Cummings BC, Colmenero CI, Rygalski CJ, Hsu CH, et al. Use of resuscitative balloon occlusion of the aorta in a swine model of prolonged cardiac arrest. Resuscitation. 2019;140:106–12. doi: 10.1016/j.resuscitation.2019.05.010 31121206 PMC7157798

[22] Dogan EM, Hörer TM, Edström M, Martell EA, Sandblom I, Marttala J, et al. Resuscitative endovascular balloon occlusion of the aorta in zone I versus zone III in a porcine model of non-traumatic cardiac arrest and cardiopulmonary resuscitation: a randomized study. Resuscitation. 2020;151:150–6. doi: 10.1016/j.resuscitation.2020.04.011 32339599

[23] Olsen MH, Olesen ND, Karlsson M, Holmlöv T, Søndergaard L, Boutelle M, et al. Randomized blinded trial of automated REBOA during CPR in a porcine model of cardiac arrest. Resuscitation. 2021;160:39–48. doi: 10.1016/j.resuscitation.2021.01.010 33482264

[24] Xu J, Khan ZU, Zhang M, Wang J, Zhou M, Zheng Z, et al. The combination of chest compression synchronized ventilation and aortic balloon occlusion improve the outcomes of cardiopulmonary resuscitation in swine. Front Med. 2022;9. doi: 10.3389/fmed.2022.1057000 PMC9810756 36619612

[25] Kill C, Hahn O, Dietz F, Neuhaus C, Schwarz S, Mahling R, et al. Mechanical ventilation during cardiopulmonary resuscitation with intermittent positive-pressure ventilation, bilevel ventilation, or chest compression synchronized ventilation in a pig model. Crit Care Med. 2014;42(2):e89–95. doi: 10.1097/CCM.0b013e3182a63fa0 24158168

[26] Chudeau N, Saulnier P, Parot-Schinkel E, Lascarrou J-B, Colin G, Barbar SD, et al. Mean arterial pressure after out-of-hospital cardiac arrest (METAPHORE): study protocol for a multicentre controlled trial with blinded primary outcome assessor. BMJ Open. 2025;15(4):e096997. doi: 10.1136/bmjopen-2024-096997 40280607 PMC12035465

[27] Koyama Y, Matsuyama T, Inoue Y. Association between haemodynamics during cardiopulmonary resuscitation and patient outcomes. Resuscitation. 2022;170:295–302. doi: 10.1016/j.resuscitation.2021.10.019 34673153

[28] Maldonado FA, Weil MH, Tang W, Bisera J, Gazmuri RJ, Johnson B, et al. Myocardial hypercarbic acidosis reduces cardiac resuscitability. Anesthesiology. 1993;78(2):343–52. doi: 10.1097/00000542-199302000-00019 8439030

[29] Lin C-H, Yu S-H, Chen C-Y, Huang F-W, Chen W-K, Shih H-M. Early blood pH as an independent predictor of neurological outcome in patients with out-of-hospital cardiac arrest: a retrospective observational study. Medicine (Baltimore). 2021;100(17):e25724. doi: 10.1097/MD.0000000000025724 33907164 PMC8084093

[30] Dogan EM, Dogan EA, Nilsson KF, Edström M. Intra-aortic balloon pump synchronized with chest compressions improves outcome during cardiopulmonary resuscitation in experimental cardiac arrest. Resuscitation. 2024;205:110433. doi: 10.1016/j.resuscitation.2024.110433 39542127

[31] Low GKK, Azahar A, Samson E, Rane P. Systematic review of swine models for ventricular fibrillation induction in evaluating cardiopulmonary resuscitation methods. Cardiology Plus. 2024;9(2):91–102. doi: 10.1097/cp9.0000000000000087

[32] Brede JR, Lafrenz T, Klepstad P, Skjærseth EA, Nordseth T, Søvik E, et al. Feasibility of pre-hospital resuscitative endovascular balloon occlusion of the aorta in non-traumatic out-of-hospital cardiac arrest. J Am Heart Assoc. 2019;8(22):e014394. doi: 10.1161/JAHA.119.014394 31707942 PMC6915259

[33] Brede JR, Skulberg AK, Rehn M, Thorsen K, Klepstad P, Tylleskär I, et al. REBOARREST, resuscitative endovascular balloon occlusion of the aorta in non-traumatic out-of-hospital cardiac arrest: a study protocol for a randomised, parallel group, clinical multicentre trial. Trials. 2021;22(1):511. doi: 10.1186/s13063-021-05477-1 34332617 PMC8325811

